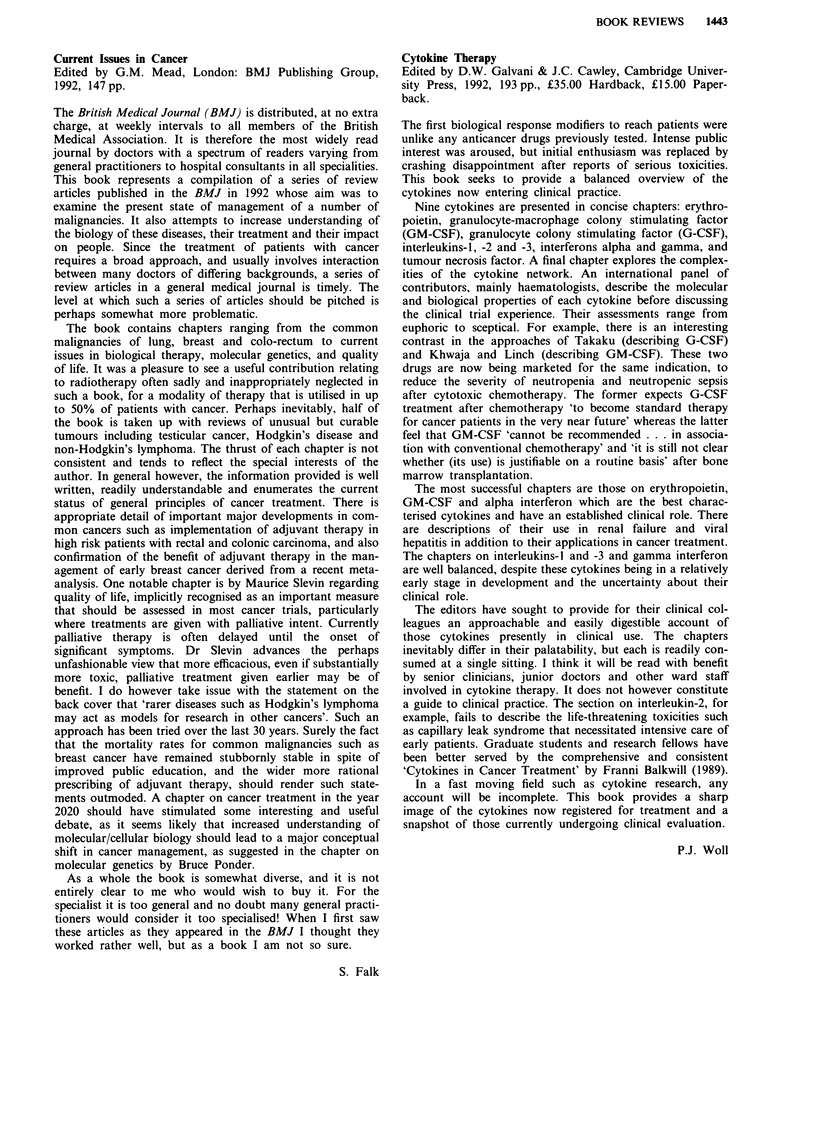# Current Issues in Cancer

**Published:** 1993-06

**Authors:** S. Falk


**Current Issues in Cancer**
Edited by G.M. Mead, London: BMJ Publishing Group, 1992, 147 pp.

The *British Medical Journal (BMJ)* is distributed, at no extra charge, at weekly intervals to all members of the British Medical Association. It is therefore the most widely read journal by doctors with a spectrum of readers varying from general practitioners to hospital consultants in all specialities. This book represents a compilation of a series of review articles published in the *BMJ* in 1992 whose aim was to examine the present state of management of a number of malignancies. It also attempts to increase understanding of the biology of these diseases, their treatment and their impact on people. Since the treatment of patients with cancer requires a broad approach, and usually involves interaction between many doctors of differing backgrounds, a series of review articles in a general medical journal is timely. The level at which such a series of articles should be pitched is perhaps somewhat more problematic.

The book contains chapters ranging from the common malignancies of lung, breast and colo-rectum to current issues in biological therapy, molecular genetics, and quality of life. It was a pleasure to see a useful contribution relating to radiotherapy often sadly and inappropriately neglected in such a book, for a modality of therapy that is utilised in up to 50% of patients with cancer. Perhaps inevitably, half of the book is taken up with reviews of unusual but curable tumours including testicular cancer, Hodgkin's disease and non-Hodgkin's lymphoma. The thrust of each chapter is not consistent and tends to reflect the special interests of the author. In general however, the information provided is well written, readily understandable and enumerates the current status of general principles of cancer treatment. There is appropriate detail of important major developments in common cancers such as implementation of adjuvant therapy in high risk patients with rectal and colonic carcinoma, and also confirmation of the benefit of adjuvant therapy in the management of early breast cancer derived from a recent meta-analysis. One notable chapter is by Maurice Slevin regarding quality of life, implicitly recognised as an important measure that should be assessed in most cancer trials, particularly where treatments are given with palliative intent. Currently palliative therapy is often delayed until the onset of significant symptoms. Dr Slevin advances the perhaps unfashionable view that more efficacious, even if substantially more toxic, palliative treatment given earlier may be of benefit. I do however take issue with the statement on the back cover that 'rarer diseases such as Hodgkin's lymphoma may act as models for research in other cancers'. Such an approach has been tried over the last 30 years. Surely the fact that the mortality rates for common malignancies such as breast cancer have remained stubbornly stable in spite of improved public education, and the wider more rational prescribing of adjuvant therapy, should render such statements outmoded. A chapter on cancer treatment in the year 2020 should have stimulated some interesting and useful debate, as it seems likely that increased understanding of molecular/cellular biology should lead to a major conceptual shift in cancer management, as suggested in the chapter on molecular genetics by Bruce Ponder.

As a whole the book is somewhat diverse, and it is not entirely clear to me who would wish to buy it. For the specialist it is too general and no doubt many general practitioners would consider it too specialised! When I first saw these articles as they appeared in the *BMJ* I thought they worked rather well, but as a book I am not so sure.

S. Falk